# Effective field of view of wide-field fundus photography in the Stanford University Network for Diagnosis of Retinopathy of Prematurity (SUNDROP)

**DOI:** 10.1038/s41598-022-22964-w

**Published:** 2022-11-11

**Authors:** Marco H. Ji, Moosa Zaidi, Zachary Bodnar, Sean K. Wang, Jochen Kumm, Darius M. Moshfeghi

**Affiliations:** 1 Department of Ophthalmology, Jones Eye Institute, University of Arkansas for Medical Sciences, Little Rock, AR USA; 2 Department of Ophthalmology, Horngren Family Vitreoretinal Center, Byers Eye Institute, Stanford University School of Medicine, 2452 Watson Court, Rm. 2277, Palo Alto, CA 94303 USA; 3 Meadows Eye Physicians and Surgeons, Las Vegas, NV USA; 4 Pr3vent Inc, Palo Alto, CA USA

**Keywords:** Retinopathy of prematurity, Retinal diseases

## Abstract

Five-field 130° wide-angle imaging is the standard of care for retinopathy of prematurity (ROP) screening with an ideal hypothetical composite field-of-view (FOV) of 180°. We hypothesized that in many real-world scenarios the effective composite FOV is considerably less than ideal. This observational retrospective study analyzed the effective FOV of fundus photos of patients screened for ROP as part of the Stanford University Network for Diagnosis of Retinopathy of Prematurity (SUNDROP) initiative. Five fundus photos were selected from each eye per image session. Effective FOV was defined as the largest circular area centered on the optic disc that encompassed retina in each of the four cardinal views. Seventy-three subjects were analyzed, 35 without ROP and 34 with ROP. Mean effective FOV was 144.55 ± 6.62° ranging from 130.00 to 153.71°. Effective FOV was not correlated with the presence or absence of ROP, gestational age, birth weight, or postmenstrual age. Mean effective FOV was wider in males compared to females. Standard five-field 130° fundus photos yielded an average effective FOV of 144.54° in the SUNDROP cohort. This implies that an imaging FOV during ROP screening considerably less than the hypothetical ideal of 180° is sufficient for detecting treatment warranted ROP.

## Introduction

Retinopathy of prematurity (ROP) is a sight-threatening disease that can affect preterm infants and is one of the leading causes of childhood blindness in both developed and developing countries^1,2^. The American Academy of Ophthalmology (AAO) and the American Academy of Pediatrics (AAP) guidelines recommend that infants with gestational age (GA) 30 weeks or less, birth weight (BW) 1500 g or less, or a complicated clinical course be screened for ROP using indirect ophthalmoscopy by an ophthalmologist. The first exam is recommended at 31 weeks for infants with GA of 27 weeks or less and at 4 weeks of chronological age for infants with GA of 27 weeks or older^3^. Although binocular indirect ophthalmoscopy (BIO) remains the gold standard, recent developments in ophthalmic imaging have expanded the tools available for ophthalmologists providing ROP care. In particular, the introduction of portable wide-field 130° fundus cameras such as the Retcam (Natus Medical Inc., Pleasanton, CA, formerly Clarity Medical Systems Inc.) have allowed for telemedicine screening programs in neonatal intensive care units (NICUs) via bedside fundus photography. These telemedicine initiatives have the potential to provide high quality, accessible, and cost effective ROP care^4,5^.

The Stanford University Network for Diagnosis of Retinopathy of Prematurity (SUNDROP) is the first and largest telemedicine screening program for ROP in the United States, currently active in ten NICUs across California, Nevada, and Indiana^6–10^. The imaging probe used by SUNDROP NICUs s consists of a camera coupled with a 130° lens; the probe is tilted in multiple directions to capture the peripheral retina. Current treatment triggers for ROP occur in Zone I or Zone II, which should theoretically be captured by a single 130° lens but may not be due to anatomic differences and optical considerations that vary from eye to eye. This study aimed to evaluate the actual field of view (FOV) of wide-field fundus cameras by assessing the effective composite FOV obtained by all images in each imaging session.

## Methods

This study was conducted in accordance with the Health Insurance Portability and Accountability Act (HIPAA) and the tenets of the Declaration of Helsinki, and approval was given by the Institutional Board Review (IRB 8752) at Stanford University School of Medicine, which granted a waiver of informed consent for retrospective data analysis of efficacy and outcomes.

All infants who met the abovementioned screening criteria for ROP at the ten SUNDROP sites underwent telemedicine examination using a Retcam II or III camera coupled with a wide-field 130° FOV lens and were included in the SUNDROP database. All images were taken by trained nursing staff and transferred using secure end-to-end encrypted email or uploaded to RetCam Review Software. For this retrospective study we selected randomly five subjects per year among infants enrolled in the SUNDROP initiative that underwent screening between January 1, 2006 and December 31, 2019. For each subject both eyes from one single random session were evaluated in our study. Each exam session consisted of six standard photographs per eye that captured the iris, posterior pole, superior, inferior, temporal, and nasal quadrants for a total of 12 images per session. For this study, iris photos were excluded from the analysis, resulting in five fundus photos per eye.

Effective FOV was defined as the largest area of a circle centered on the optic disc that encompassed retina in each of the four cardinal views. Because images were initially labeled by subject but not laterality, laterality was inferred for each image by visual inspection. The baseline FOV radius, corresponding to the reported 130° FOV, was computed for each eye as half the pixel count along the shorter dimension of images recorded for that eye. The approximate location of the optic disc within each image was marked in each image. Images for which poor quality precluded estimation of the optic disc location were excluded at this step. For images in which the optic disc appeared to be outside the field of view, if the direction of the optic disc could be reasonably inferred, then the optic disc location was marked as the center of the image edge most in the direction of the disc; otherwise, if optic disc location could not be reasonably inferred, then the image was excluded.

**Figure 1:**
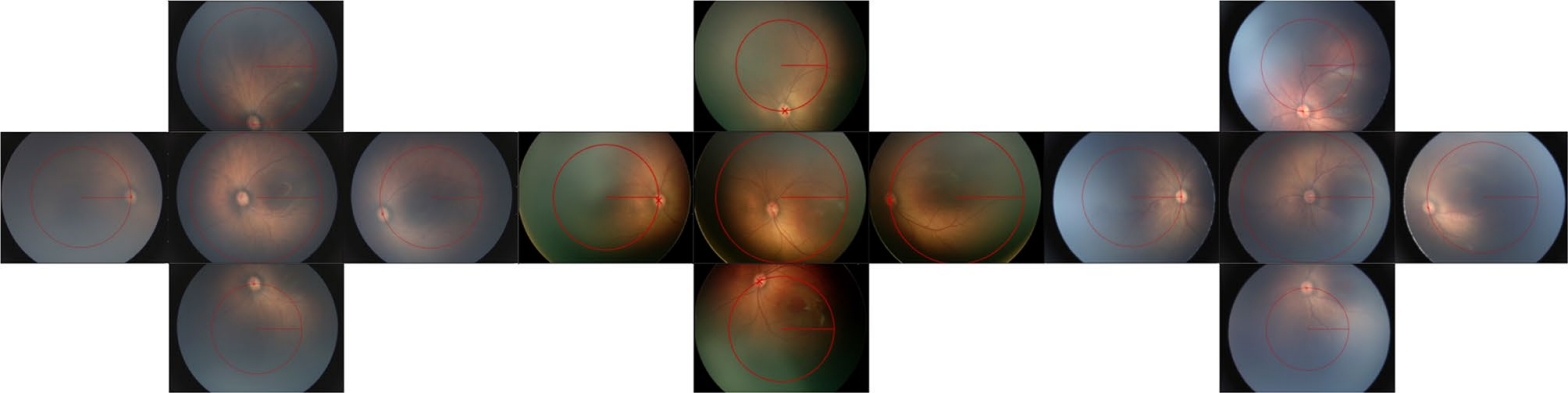
Illustration of how the radius in each photo of a session was measured. The red X represents the optic disc marked as reference for the measurements.

For each eye, the marked optic disc locations were then used to estimate the effective FOV radius in each cardinal direction (superior, inferior, temporal, nasal) relative to the optic disc center (Fig. 1). For example, to determine the effective FOV radius in the superior direction, the distance from image center to the marked disc location was computed for all nerve inferior images of that eye. An image was considered superior for the purpose of this calculation if the disc was inferior to the image center and if the superior-inferior component of the vector from the image center to the disc was at least half the nasal-temporal component. The maximum image center to disc location distance of any superior image (defaulting to zero if no superior images were present) was added to the baseline FOV radius computed in the first part of this analysis to arrive at the effective FOV radius in the superior direction. The effective FOV radius in each of the other three cardinal directions was analogously computed. The minimum effective FOV radius across the four cardinal directions was taken to be the overall effective FOV radius. Because different generations of Retcam with different resolutions were used across the years and various institutions, measurements made on images taken using the newer IDS 1490 image sensor were converted using a multiplier of 0.4 to make them comparable to images taken with the older Toshiba sensor. Finally, each effective FOV radius was converted to the equivalent degrees of FOV using $2 \cdot {\text{arctan}}\left( {r/b \cdot {\text{tan}}\left( {\left( {130^\circ } \right)/2} \right) } \right))$, where *r* was the effective FOV radius and *b* was the baseline FOV radius. Statistical analyses were performed using IBM SPSS Statistics (IBM Corporation, Armonk, NY). Independent Student’s T-Test, Paired Student T-Test, Chi-square and Pearson’s correlation were applied depending on the distribution of the data.

### Conference presentation

Presented as poster at the Association for Research in Vision and Ophthalmology 2020, Baltimore, MD.

## Results

To estimate the effective FOV achievable using the RetCam coupled with a 130° lens, 1,257 images taken from 73 subjects (73 right eyes, and 71 left eyes) were retrospectively analyzed. A small number of images (33/1257) were excluded because laterality could not be determined due to poor image quality or due to inability to infer optic disc location. 41 male and 32 female infants, 35 without ROP and 38 with ROP were enrolled in this study. Mean GA was 28.04 ± 2.31 (range 24–32) weeks, mean BW was 1089.79 ± 349.26, and mean postmenstrual age (PMA) at the time of the exam 36.07 ± 3.2 (range 31–44) weeks (Table 1). The temporal FOV was the largest with an average of 136.1° from the optic disc, followed by superior FOV with 102.2°, inferior with 90.3° and nasal with 80.3°.

**Table 1:** Demographics.

Male/Female	41/32
ROP/no ROP	38/35
GA (mean ± SD) weeks	28.04 ± 2.31
BW (mean ± SD) g	1089.79 ± 349.26
PMA (mean ± SD) weeks	36.07 ± 3.2

The mean effective FOV was 144.55 ± 6.62°—specific images sets had effective FOV ranging from 130.00 to 153.71° (Table 2). There was no significant difference between patients with ROP and without ROP (*p* = 0.15). Effective FOV was not correlated with GA (*p* = 0.996), BW (*p* = 0.116), or PMA at the time of the exam (*p* = 0.305). Mean effective FOV was larger in males (146.16 ± 5.60°) compared to females (142.48 ± 7.27°) (*p* = 0.001), although there was no inter-sex difference in BW (*p* = 0.99), GA (*p* = 0.44), ROP (*p* = 0.704), or PMA(*p* = 0.856). An additional sub-analysis showed that in males the FOV was statistically larger compared to females in three of the four quadrants (*p* < 0.05), but not in the inferior view (*p* = 0.40).

**Table 2:** Field of views.

Superior FOV	102.18 ± 31.58
Inferior FOV	90.29 ± 34.01
Nasal FOV	80.30 ± 33.13
Temporal FOV	136.09 ± 35.10
Effective FOV	144.55 ± 6.62

Paired t-tests between the two eyes showed no consistent effective of laterality on FOVs (*p* = 0.70). However, when each specific view was compared, the left eye superior view had an additional mean 8.0° compared to the right eye superior view, although statistical significance was not reached (*p* = 0.067).

## Discussion

In the last two decades, fundus photography in the management of ROP has been increasingly adopted in routine clinical settings as well as for research purposes. Numerous studies have supported its validity and diagnostic accuracy in the context of telemedicine with a remote physician or even trained technicians reading the images^6–12^. The documentation provided by fundus photos additionally enables direct comparison among sessions, allowing for tracking of both disease progression^13^ and treatment response^14,15^.

Ells et al. introduced the first standardized protocol for telemedicine-based ROP screening consisting of five images per eye including the posterior pole, temporal, nasal, superior, and inferior retina^16^. This protocol has since been used worldwide in both clinical and research settings, allowing for comparisons within and across different studies^17,18^. The fully developed adult retina covers 72% of the inner ocular globe^19^, corresponding to roughly to 180° of visual field per eye^20^. Although the retinal periphery cannot be fully imaged using this protocol, real-world data from the SUNDROP initiative revealed that no treatment warranted -ROP (TW-ROP) cases were missed (sensitivity = 1.0) over more than 1,800 screened infants to date^10^. Our study showed that when this standardized protocol is used the effective FOV is on average 144.54°, only 14.54° (11%) more than the single image of the posterior pole with a 130° view. Nonetheless, the FOV provided by this average effective FOV of 144.54° was sufficient to detect all clinically relevant findings. Together, our data suggest that a single photo centered on the optic nerve from a hypothetical 145° lens might be sufficient to rule out TW-ROP. Conversely, we hypothesize that a system with a narrower FOV that takes multiple photos at different angles might be sensitive enough if the composite of photos can reach an effective FOV of 145° or higher.

In this study we demonstrated that the FOV was not symmetrically distributed around the optic nerve, with the temporal FOV being the widest followed by superior, inferior, and nasal fields. The optic nerve head was used as reference because it is easily detectable in the posterior pole. However, the optic nerve lies nasally compared to the intersection between the pupillary axis and the retinal plane. At the same time, ROP often presents with a vascular wedge on the temporal retina with the apex pointing to the optic disc^21^. As a consequence, the temporal view is both the widest and the highest yield for detecting disease.

The average FOV between the two eyes was similar in this study. Although not a statistically significant difference, the superior quadrant of the right eye tended to have a more limited average view compared to the left eye. Considering that most individuals are right-handed, this small discrepancy might be explained by different ergonomics of the probe on the two ocular globes. The average effective FOV of infants with ROP was comparable to that of patients without ROP. This suggests that all infants were equally imaged without performance bias towards sicker patients. Also, no correlation was found between effective FOV and GA, BW, or PMA.

We found that the effective FOV was wider in males compared to females in every quadrant except the inferior view despite comparable GA, BW, PMA, and ROP status. Previous studies have shown that males have longer eyes during the neonatal stage even after correcting for BW and GA^22,23^. This greater axial length might correlate with a larger retina as well as a larger pupillary aperture that provides a wider view of the fundus periphery. Additionally, there might have been different efficacies for the mydriatic drops between the two sex groups due to ethnic imbalances since eyes with dark irises are less responsive to mydriatics^24,25^.

To summarize, five multi-directional fundus photos acquired by a 130° camera in a real-world setting yielded an average effective FOV of 144.54°. This was less than the assumed 180° FOV but was sufficient to detect 100% of the TW-ROP in the SUNDROP cohort in more than 1,800 screened infants to date. This implies that an imaging FOV during ROP screening considerably less than the hypothetical ideal of 180 is sufficient for patient safety. Males were found to have a larger effective FOV compared to females. Additional studies are warranted to answer the questions that our study raised and to further validate the use of fundus photography in the management of ROP.

## Data Availability

The datasets used and/or analysed during the current study available from the corresponding author on reasonable request.
